# Expressive Timing Facilitates the Neural Processing of Phrase Boundaries in Music: Evidence from Event-Related Potentials

**DOI:** 10.1371/journal.pone.0055150

**Published:** 2013-01-30

**Authors:** Eva Istók, Anders Friberg, Minna Huotilainen, Mari Tervaniemi

**Affiliations:** 1 Cognitive Brain Research Unit, Cognitive Science, Institute of Behavioural Sciences, University of Helsinki, Helsinki, Finland; 2 Finnish Centre of Excellence in Interdisciplinary Music Research, Department of Music, University of Jyväskylä, Jyväskylä, Finland; 3 Department of Speech, Music, and Hearing, KTH Royal Institute of Technology, Stockholm, Sweden; 4 Finnish Institute of Occupational Health, Helsinki, Finland; 5 Department of Psychology, University of Jyväskylä, Jyväskylä, Finland; Baycrest Hospital, Canada

## Abstract

The organization of sound into meaningful units is fundamental to the processing of auditory information such as speech and music. In expressive music performance, structural units or phrases may become particularly distinguishable through subtle timing variations highlighting musical phrase boundaries. As such, expressive timing may support the successful parsing of otherwise continuous musical material. By means of the event-related potential technique (ERP), we investigated whether expressive timing modulates the neural processing of musical phrases. Musicians and laymen listened to short atonal scale-like melodies that were presented either isochronously (deadpan) or with expressive timing cues emphasizing the melodies’ two-phrase structure. Melodies were presented in an active and a passive condition. Expressive timing facilitated the processing of phrase boundaries as indicated by decreased N2b amplitude and enhanced P3a amplitude for target phrase boundaries and larger P2 amplitude for non-target boundaries. When timing cues were lacking, task demands increased especially for laymen as reflected by reduced P3a amplitude. In line, the N2b occurred earlier for musicians in both conditions indicating general faster target detection compared to laymen. Importantly, the elicitation of a P3a-like response to phrase boundaries marked by a pitch leap during passive exposure suggests that expressive timing information is automatically encoded and may lead to an involuntary allocation of attention towards significant events within a melody. We conclude that subtle timing variations in music performance prepare the listener for musical key events by directing and guiding attention towards their occurrences. That is, expressive timing facilitates the structuring and parsing of continuous musical material even when the auditory input is unattended.

## Introduction

The segmentation of auditory information such as music and spoken language into meaningful units is central to human information processing. It guides our perception and facilitates the organization of sounds [1]. In music, units or phrases often occur regularly (e.g., every four or eight bars) and are marked by pauses, pitch leaps, harmonic or melodic changes. Beyond those compositional characteristics, it is the performer who may actively influence the way how the audience perceives the music by slightly varying those acoustic parameters that provide cues necessary for the successful structuring of the musical input.

Besides micro-variations in pitch, sound level, articulation, timbre, and tempo, performers frequently modulate the timing of successive musical events to bring out the structure of the music [2]. This so-called expressive timing specifically aids the sequential organization of musical phrases. Similar to the phrase-final lengthening in spoken language, performers tend to slow down tempo and to diminish the sound level at the end of a musical phrase [3]. Expressive timing cues provide the listener not only with information about the upcoming closing of a musical phrase, but they also prepare for the occurrence of a new important musical event, such as the beginning of a new phrase. In the present study, we investigated how such subtle timing variations affect the neural processing of phrase boundaries by means of the event-related potential technique (ERP).

From a neural perspective, it is of crucial interest which mechanisms are involved in the segmentation of music and speech. First, acoustic cues marking a phrase boundary must be detected and recognized. Second, the successful identification of these cues might generate expectations about the continuation of the music. Finally and most importantly, expectations might trigger the allocation of attention towards the phrase boundary and hence facilitate its perceptual processing.

Because of its high temporal resolution, measuring the electroencephalogram and quantifying brain responses using the ERP is specifically suited to study the timing and the nature of those perceptual and cognitive processes that underlie our capability to extract important events from the auditory environment.

Several components of the ERP have been linked to sound discrimination and recognition, expectation and attention allocation in general and to the processing of phrase boundaries in music and speech in particular. These will be introduced in the following.

The auditory P2 is commonly associated with the perceptual processing of acoustic features, stimulus identification and classification, although its functional meaning is not well specified (for a review of the P2 component see [4]). The P2 typically peaks between 150 and 250 ms and shows a fronto-central scalp maximum (e.g., [5]). It usually occurs in combination with the N1 component (N1-P2 complex) and may be elicited by both attended and ignored stimuli (e.g., [6]).

In contrast, conscious sound discrimination, target detection and attention allocation have been associated with the N2b-P3a complex of the ERP [7]. The N2b shows a posterior distribution [8] and has specifically been linked to the controlled orienting towards a deviant sound occurring in otherwise regular sound sequences [9]. The amplitude of the N2b has been shown to be larger for unexpected compared to more expected target events [10] and to be reduced when sound discrimination becomes more difficult [11].

The P3a is considered to be a sub-process of the P300 (P3) component. It is maximal over frontal regions and has been associated with focal attention needed to process novel or unexpected stimulus events. The P3a amplitude is sensitive to task demands: difficult tasks elicit smaller P3a amplitude compared to less demanding tasks (for a review of the P3a component, see [12]).

The processing of phrase boundaries in music has been associated with the P2 component and a positive deflection in the ERP labeled music closure positive shift (‘music CPS’) [13],[14],[15]. Analogue to the language CPS [16],[17] which seemingly reflects closure processes rather than the processing of prosodic cues per se, the ‘music CPS’ is believed to be a sign of memory and attention processes needed to guide the perception from one musical phrase to the other. The neural processing of phrase boundaries in music was shown to be sensitive to the pause length preceding the phrase boundary and to the harmonic context, and, hence, to structural music features [13],[15]. Yet, it remains to be determined whether performance expression similarly modulates brain indices of phrase boundary processing in music.

Therefore, the present study aimed at specifically studying possible effects of expressive timing on phrase boundary processing. Phrasing patterns in Western tonal music may be determined by their tonal and harmonic structure that has been shown to also affect the neural processing of phrase boundaries in music [15]. To single out the effects of expressive timing and to control for possibly confounding effects of tonal expectations, we decided to use atonal scale-like melodies consisting of two phrases as experimental material. The melodies were presented either isochronously or with expressive timing variations emphasizing the two-phrase structure. Based on the assumption that expressive timing generates expectations about the musical structure and guides listeners’ attention towards significant events within the music, we quantified ERP responses to the beginning note of the second phrase, hence, to a significant musical event. In half of the melodies, the beginning of the second phrase was marked by a pitch leap that served as target for a phrase boundary detection task.

We hypothesized that the conscious detection of phrase boundaries would be reflected by the occurrence of an N2b-P3a complex in the ERP. We expected the N2b amplitude to be reduced for the expressive timing condition reflecting facilitated processing of the phrase boundary. We further assumed that the P3a would be reduced in those conditions where no timing information was provided, because the lack of timing cues increased task demands.

Moreover, we hypothesized that a music CPS would be elicited by phrase boundaries that were only marked by variations in timing patterns but not by an additional pitch leap (non-target boundaries), because previous studies [13],15] found the CPS when participants actively listened to phrased melodies without paying attention to the phrase boundary (i.e., non-targets).

Because earlier studies have shown that sound discrimination abilities are superior in musicians compared to laymen [18], we assumed that the detection of phrase boundaries would be more difficult for laymen compared to musicians, especially for deadpan melodies. Accordingly, we expected that the N2b would be larger and the P3a smaller for laymen compared to musicians.

Although top-down effects, such as attention may influence the segmentation of our auditory environment, there is ample evidence that sound organization to a great extent takes place automatically [19]. In fact, already Gestalt theory [20] postulated that processes of auditory grouping are automatic in nature. Later, this view has been further promoted [1]. Based on these theories, we tested whether phrase boundary processing as reflected in the P2 and CPS components would occur also when the melodies were unattended. We therefore presented the musical material during a passive and an active condition.

## Methods

### Ethics Statement

The study was approved by the ethical committee of the former Department of Psychology, University of Helsinki, and all participants gave written informed consent prior to the experiment.

### Participants

In total, 20 participants (mean age = 24.8 years; *SD* = 3.34; 10 females) completed the experiment. All volunteers reported normal hearing. Ten of the participants were professional or semi-professional musicians (mean age = 25.7 years; *SD* = 3.40; 5 females), and 10 did not have a special musical training (mean age = 24.0 years; *SD* = 3.24; 5 females).

None of the participants was taking medication affecting the central nervous system and none of them reported on neurological problems. Handedness was tested with a Finnish version of the Edinburgh Handedness Inventory. Two of the participants were left-handed. All participants received a modest financial compensation.

### Materials

The stimulus set was created out of six scale-like melodies consisting of equal interval steps: ascending minor seconds, descending minor seconds, ascending major seconds, descending major seconds, ascending minor thirds, or descending minor thirds. A pitch leap larger than the basic interval was inserted in the middle of the melodies to mark the beginning of the second phrase, hence, the phrase boundary. To vary the difficulty level, the size of the pitch leap ranged from three semitones (minor third) to nine semitones (major sixth). For ascending and descending melodies consisting of minor seconds, the pitch leap size was a minor third, a major third, a perfect forth, or diminished fifth. For melodies consisting of major seconds, the pitch leap size was a perfect forth, diminished fifth, perfect fifth, or minor sixth. Finally, for melodies made up of minor thirds the pitch leap size was a diminished fifth, perfect fifth, minor sixth, or major sixth.

**Figure 1:**
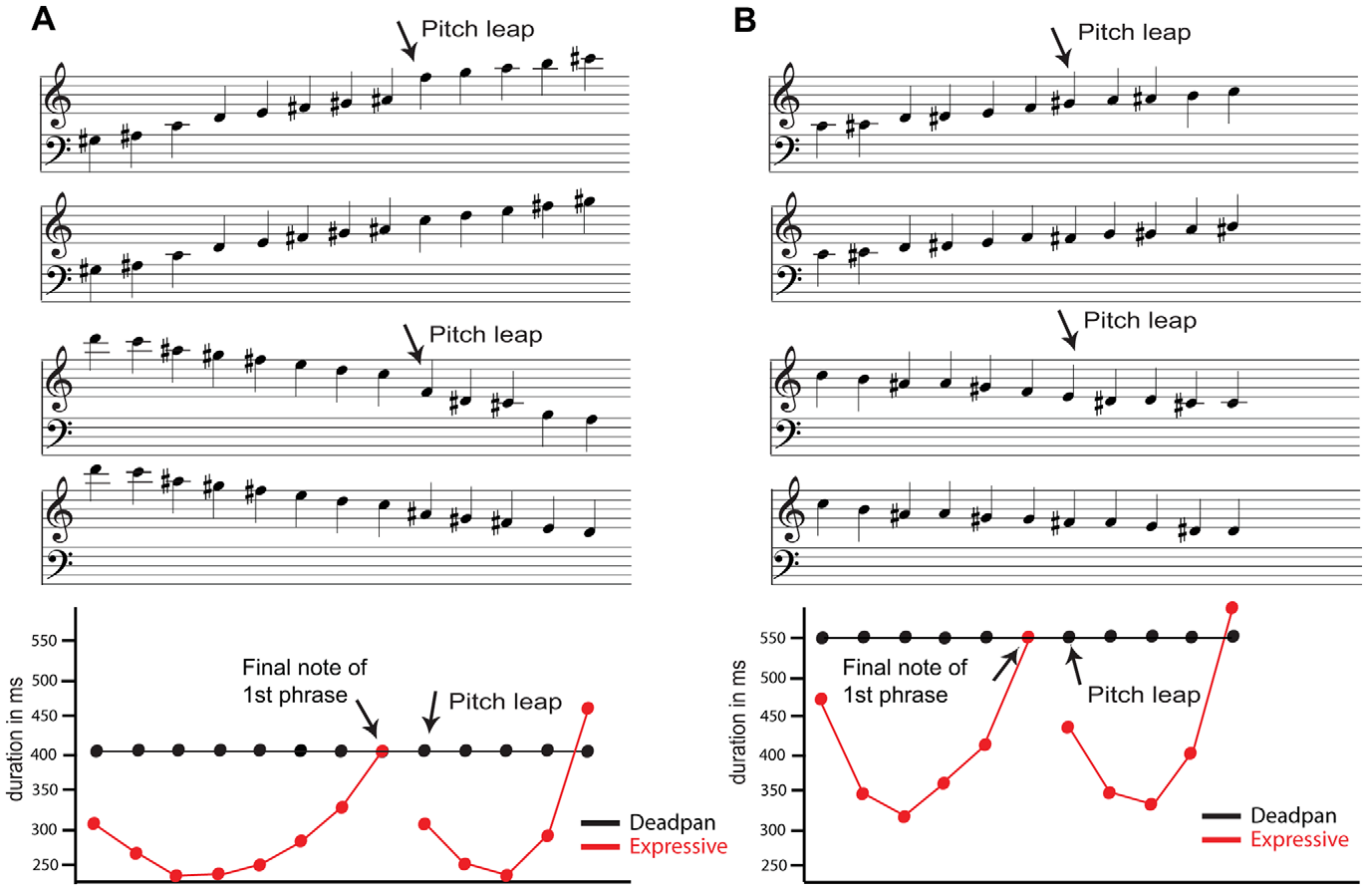
Examples of the stimulus material. A) Ascending and descending melodies consisting of major seconds with and without pitch leap. The pitch leap size here is a perfect fifth. The lower panel shows the duration of each note in ms for deadpan (black) and expressive versions (red) of the examples above at tempo 150 bpm. B) Ascending and descending melodies consisting of minor seconds with and without pitch leap. The pitch leap size here is a minor third. The lower panel shows the duration of each note in ms for deadpan (black) and expressive versions (red) of the examples above at tempo 109 bpm.

To avoid that our participants anticipated the occurrence of the boundary, four versions were created for each of the six basic melodies by inserting the pitch leap at one of four different positions within the melodies (after five, six, seven or eight tones). The second phrase always consisted of five tones starting with the leap. Thus, depending on the position of the pitch leap the melodies were composed of 11 to 13 tones. Figure 1A and 1B show example melodies with and without pitch leap. The resulting 24 melodies were transposed up (ascending melodies) or down (descending melodies) one, two, and three semitones together amounting to a set of 96 melodies. The size of the inserted pitch leap differed between the transpositions. The pitch leap increased with up transpositions and down transpositions for ascending and descending melodies respectively. The melodies were presented with 150, 126 or 109 beats per minute (bpm) to control for tempo effects. Of each of the 96 melodies, one version without a pitch leap was created by replacing the leap with the basic interval of the melody. Hence, these additional 96 melodies consisted of 11 to 14 ascending or descending equal interval steps (minor seconds, major seconds, or minor thirds). Finally, two versions of the resulting 192 melodies were generated: a deadpan version with isochronous tone onsets and an expressive version where the timing of the individual notes was modified. The complete set of melodies contained 384 examples.

The expressive phrasing patterns of the melodies were generated using Director Musices (v. 2.7), a software package developed at the KTH Royal Institute of Technology, Stockholm, Sweden. The program supports the transformation of notated scores into music performances and is based on a generative rule system [21]. This rule system includes, for example, rules for phrasing, timing, articulation, and intonation patterns which all can be applied gradually (for an overview, see [22]). To modify the timing of the phrasing patterns, we applied the so-called phrase arch rule [23]. The rule modifies the local tempo and dynamics of each phrase using an arch-like shape. That is, the beginning of the phrase is slow and soft, the middle of the phrase is faster and more intense, and the end of the phrase is again slow and soft. Because we aimed at investigating only the timing effects on the detection of phrase boundaries, we kept the dynamic level constant in this case. The phrase arch rule was applied with the quantity 3.5 (for explanation, see [23]), the turning position occurred at 35% of the total phrase length with a parabolic shape (see Figure 1, lower panel). We used an identical grouping structure for both types of melodies (with pitch leap, without pitch leap) to compute the phrase arch. That is, melodies with and without pitch leap contained the same expressive timing structure.

The scores were automatically generated in Director Musices using a custom script considering all the different combinations of scales and leaps described above. Importantly, we controlled for possible effects of neural recovery by adjusting the expressive versions in tempo so that the length of the final note in the first phrase was the same as in the corresponding deadpan version. Thus, the inter onset interval (IOI) preceding the phrase boundary was identical for the two conditions.

This had the side effect that the overall tempo of the excerpt was slightly different for the expressive version compared to the deadpan version. This effect was considered negligible because the resulting spread in each condition due to the different initial tempi was considerably larger than the tempo alterations caused by adjusting the IOI.

Finally, all performances were saved as MIDI files. The resulting files were then converted into audio files (stereo, 44.1 kHz sampling rate) using high quality recordings of a Steinway grand piano provided in the Kontakt 2 sampler (Native Instruments). The piano sampling was manually controlled and further adjusted so that the dynamic level over the pitch range was constant.

### Procedure

The experiment consisted of two different sessions (active and passive) that took place on different days. Each participant first completed the passive before the active part. There was a break of at least one week between the sessions.

### Passive Session

In the beginning of the passive session, each participant filled in an informed consent form. Thereafter, written and oral instructions regarding the experimental procedure were given. Participants were told that they were going to watch a silent movie of their choice with subtitles while different melodies were going to be presented via headphones. Participants were asked to ignore the music and to concentrate on the movie.

The complete set of 384 melodies was presented twice. The melodies were randomly played in eight different blocks each consisting of 96 melodies. Each block lasted approximately 12 minutes.

### Active Session

Before the active session started, participants were told that they were going to actively listen to the same ascending and descending melodies previously presented during the passive session. The experimenter informed that half of the melodies consisted of equal interval steps only (minor seconds, major seconds or minor thirds) and that the other half of the melodies contained an interval within the melody that was larger than the basic interval. It was also told that some of the melodies were going to be presented with expressive timing variations and some of the melodies just as if played by a computer without any human expression. It was specified that participants’ task was to ignore the timing information and to decide whether the melody contained a pitch leap or not. To assure that the participants properly understood the task, maximally five example melodies were given. In total, the complete set of 384 melodies was presented in twelve blocks each consisting of 32 melodies.

During the melody presentation, a white fixation cross (font size: 100) was displayed on a black screen. After each melody, participants gave their answer (yes, no) by pressing one of two buttons on a response box (RB-834, Cedrus corporation, USA) which was placed on each volunteer’s lap. They were asked to use the index and middle finger of their dominant hand for their ‘yes’ and ‘no’ answers respectively. To avoid contamination of the EEG signal with motor preparatory signals, a cue (white square, 15×15 cm) appeared on the computer screen at 1000 ms after melody offset to signal that participants were allowed to answer. The cue disappeared as soon as one of the buttons was pressed and the next trial was initiated.

### Stimulus Presentation And Electrophysiological Recordings

In both sessions, the sound level of the melodies was adjusted to 40 dB above the individual subject’s hearing threshold. The musical material was presented via headphones (Sony MDR-7506 Professional). Stimulus presentation was carried out with the software programme Presentation (version 12.1 04. 10.08; Neurobehavioural Systems, Inc., Albany, CA, USA).

After the completion of the preparation for the EEG measurement, participants were comfortably seated in an electrically shielded and sound attenuated chamber in front of a computer monitor. Before the sequence of melodies was started, participants were told to relax and to reduce movements and blinking.

The continuous EEG was recorded with 64 Ag/AgCl electrodes mounted in nylon caps (Active Two system, Biosemi). Additional electrodes (Flat active, Biosemi) were placed at the left and right mastoid, the tip of the nose (reference), the outer canthi (horizontal electroculogram, HEOG), and above and under the right eye (vertical electrooculogram, VEOG). The sampling rate was 2048 Hz.

The passive and active recording sessions per participant lasted approximately 1 h 40 minutes and 1 h 30 minutes respectively.

### Data Analysis

The continuous EEG recordings were filtered offline between 0.5 and 40 Hz and downsampled to 256 Hz. Independent component analysis (ICA) was used for artefact removal. Only components whose generator unambiguously could be identified as eye movements were removed (at maximum two components per subject). Thereafter, the data were divided into epochs of 600 ms starting from the first note of the second phrase with a pre-stimulus baseline of 100 ms. For automatic epoch rejection a threshold of ±100 µV was used.

Averages were calculated for each individual subject separately for the four conditions: a) Deadpan melody with pitch leap; b) Expressive melody with pitch leap; c) Deadpan melody without pitch leap; d) Expressive melody without pitch leap. Thereafter, grand averages contrasting the four conditions were computed. Averaged brain responses for each condition were pooled over tempi. That is, by comparing the ERPs to deadpan versus expressive melodies, effects of tempo were cancelled out.

For both sessions, mean amplitudes were quantified for the time windows of the P2/N2b (140 to 220 ms) and CPS/P3a (300 to 400 ms). Based on the components common distribution and based on visual inspection of the grand averages, four different regions of interest (ROI) were defined for statistical analysis: left fronto-central [FC1, FC3, C1, C3, C5], right fronto-central [FC2, FC4, C2, C4, C6], left centro-parietal [CP1, CP3, CP5, P1, P3], and right centro-parietal [CP2, CP4, CP6, P2, P4].

For each of the time windows, a repeated-measures analysis of variance (ANOVA) was conducted with the factors ‘Expression’ (deadpan, expressive), ‘Leap‘ (pitch leap, no pitch leap), ‘ROI’ (left fronto-central, right fronto-central, left centro-parietal, right centro-parietal), and ‘Group’ (musicians, laymen) for the passive and active session separately. Greenhouse-Geisser correction was applied and ε values are indicated when the Mauchly test indicated a statistically significant violation of sphericity. We report original degrees of freedom for all analyses. Pairwise comparisons were based on simple main effects analysis whereby multiple comparisons were corrected using Bonferroni adjustment. All data processing was performed with custom Matlab functions and the EEGLab toolbox [24]. For statistical analysis, we used PASW Statistics 18.

## Results

### Passive Session

#### P2

**Figure 2:**
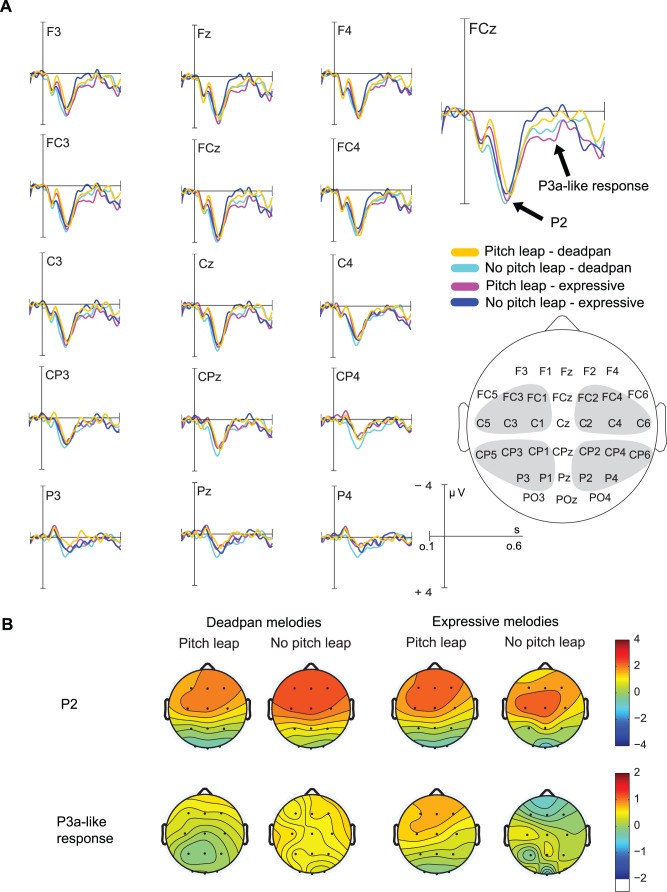
ERP results and scalp distributions during passive processing of phrase boundaries. A) Grand-average ERPs elicited by phrase boundaries (with pitch leap, without pitch leap) for expressive and deadpan melodies. The grey areas in the head model show the regions of interest (ROIs) included in the statistical analysis. A P2 in all conditions and a P3a-like response for phrase boundaries with pitch leap in expressive melodies were elicited. B) shows the scalp distributions for the P2 and P3a-like response respectively.

Figure 2 shows the grand averaged ERPs time-locked to the onset of the second phrase during the passive session. As confirmed by the main effect of ROI, *F*(3,54) = 101.5, *p*<.001, *ε* = .59, η^2^ = 0.84, a fronto-central P2 component reflecting stimulus identification was elicited in all four conditions. The P2 amplitude was smaller at right compared to left centro-parietal leads for expressive but not for deadpan melodies as indicated by the interaction effect between the factors Expression and ROI, *F*(3,54) = 4.48, *p*<.021, *ε* = .62, η^2^ = 0.19. Pairwise comparison, however, showed that the P2 amplitude differences between expressive and deadpan melodies at centro-parietal sites was not significant.

#### P3a-Like Response

When both expressive timing cues and the pitch leap together marked the onset of a new phrase, a P3a-like response was elicited between 300 and 400 ms after boundary onset. This interaction effect between Expression and Leap was significant, *F*(3,18) = 4.90, *p = *.040, η^2^ = 0.21. Simple main effects analysis revealed a main effect of Leap for expressive melodies, *F*(1,18) = 8.99, *p = *.008, but not for deadpan melodies. Generally, mean amplitudes were more positive at fronto-central compared to centro-parietal leads as indicated by the main effect of ROI, *F*(3,54) = 5.37, *p = *.008, *ε* = .69, η^2^ = 0.23.

### Active Session

#### P2/n2b

**Figure 3:**
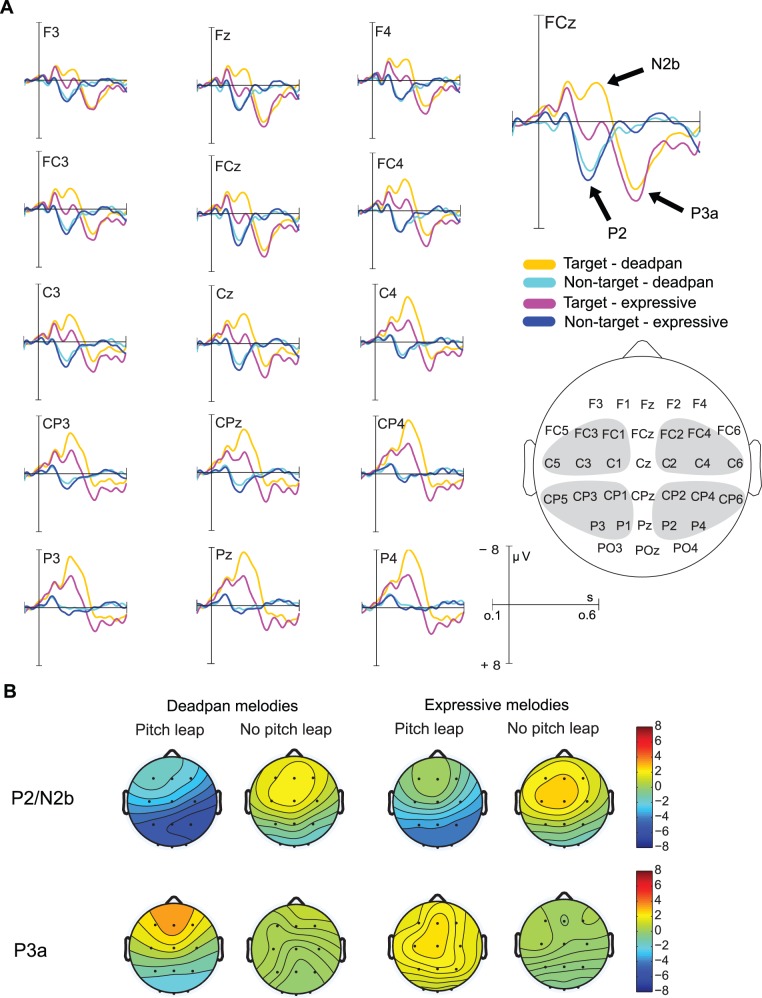
ERP results and scalp distributions during active detection of phrase boundaries. A) Grand-average ERPs elicited by phrase boundaries (with pitch leap, without pitch leap) for expressive and deadpan melodies. The grey areas in the head model show the regions of interest (ROIs) included in the statistical analysis. A P2 for non-target boundaries (without pitch leap) and a N2b and P3a for target boundaries (with pitch leap) were elicited. B) shows the scalp distributions for the P2/N2b and P3a.

**Figure 4:**
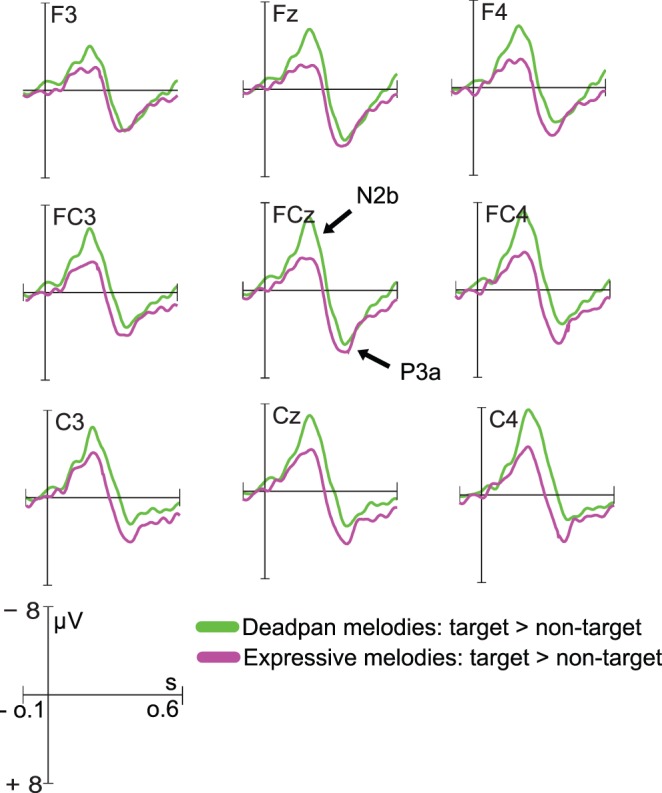
N2b/P3a difference wave. The conscious detection of phrase boundaries was indicated by the elicitation of a large N2b and P3a difference wave. N2b was reduced and P3a was enhanced for expressive melodies compared to deadpan melodies.

As illustrated in Figure 3, stimulus encoding and identification of non-target phrase boundaries was marked by a clear P2 component peaking on average at around 180 ms after phrase boundary onset for expressive and deadpan melodies. The detection of target phrase boundaries was characterized by a large N2b peaking at around 220 ms (as indicated by the difference wave shown in Figure 4). The statistical analysis confirmed this main effect of Leap, *F*(1,18) = 108.5, *p*<.001, *ε* = .69, η^2^ = 0.82. The P2 was maximal over fronto-central leads whereas the N2b was largest at posterior sites as indicated by the main effect of ROI, *F*(3,54) = 69.834, *p*<.001, *ε* = .58, η^2^ = 0.79 and the interaction effect between the factors Leap and ROI, *F*(3,54) = 5.875, *p = *.004, *ε* = .75, η^2^ = 0.24.

Both non-target stimulus encoding and active target stimulus detection as reflected by the P2 and N2b respectively were modulated by expressive timing. This was confirmed by the main effect of Expression, *F*(1,18) = 24.20, *p*<.001, η^2^ = 0.57, the interaction effect between the factors Expression and Leap, *F*(1,18) = 7.277, *p = *.015, η^2^ = 0.27, and the three-way interaction between the factors Expression, Leap, and ROI, *F*(3,54) = 3.502, *p = *.038, *ε* = .70, η^2^ = 0.16.

Pairwise comparisons revealed that the P2 amplitude was larger for expressive melodies compared to deadpan melodies, *F*(1,18) = 5.555, *p = *.030. The N2b component was reduced when the phrase boundary occurred within expressive melodies compared to its occurrence within deadpan melodies, *F*(1,18) = 21.4, *p = *.000.

**Figure 5:**
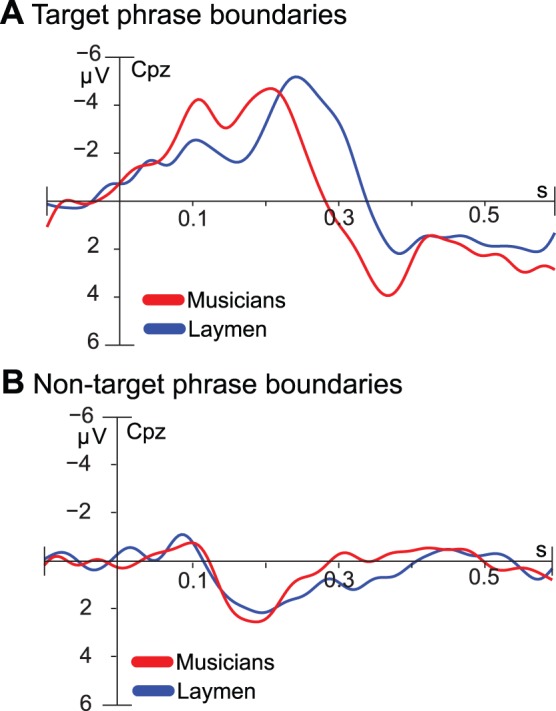
Differences between musicians and laymen. A) Grand-average ERPs (N2b/P3a) to target phrase boundaries at CPz for musicians and laymen separately. B) Grand-average ERPs at CPz reflecting the processing of non-target phrase boundaries for musicians and laymen seperately.

As Figure 5A shows, target detection was faster in musicians compared to laymen as indicated by a shorter latency of the N2b in musicians. This was confirmed by the interaction effect between the factors Leap and Group, *F*(1,18) = 6.104, *p*<.024, η^2^ = 0.05. As simple main effects analysis revealed, this effect of Group almost reached significance, *F*(1,18) = 4.316, *p*<.052. No such group difference occurred when the pitch leap was absent (Figure 5B).

#### P3a

The detection of target phrase boundaries triggered mechanisms of attention allocation as indicated by a fronto-central P3a component. This effect was confirmed by the significant interaction between the factors Leap and ROI, *F*(3,54) = 26.7, *p<*.001, *ε* = .59, η^2^ = 0.56. When no expressive timing information preceded the target phrase boundary, the P3a amplitude was reduced for deadpan compared to expressive melodies. This was confirmed by the significant main effect of Expression, *F*(1,18) = 21.2, *p*<.001, η^2^ = 0.43, and the interaction effects between Expression and Leap, *F*(1,18) = 14.4, *p = *.001, η^2^ = 0.42, between Expression and ROI, *F*(3,54) = 18.2, *p<*.001, *ε* = .71, η^2^ = 0.48, and the three-way interaction effect between the factors Expression, Leap, and ROI, *F*(3,54) = 12.0, *p<*.001, *ε* = .76, η^2^ = 0.38.

The detection of phrase boundaries within deadpan melodies seemed more demanding for laymen compared to experts as revealed by the significantly reduced P3a amplitude in laymen. This was confirmed by the interaction effect between the factors Expression and Group, *F*(1,18) = 10.1, *p = *.005, η^2^ = 0.20. Pairwise comparisons revealed a significant effect of Expression for Laymen, *F*(1,18) = 30.3, *p<*.001, but not for musicians.

#### Behavioral Results

Musicians, on average, carried out the pitch detection task correctly in 94.5% of the cases (*SD* = 5.42%), whereby the percentage of correct responses was identical for deadpan (94.4%, *SD* = 6.08%) and expressive melodies (94.5%, *SD* = 5.02%). The task performance of laymen was slightly inferior. Overall, their answers were correct in 89.6% (*SD* = 4.12%) of the melodies. The percentage of correct answers for deadpan melodies (90.5%, *SD* = 3.5%) was minimally higher compared to expressive melodies (88.7%, *SD* = 5.07%). The difference did not reach significance. The difference between musicians and laymen was significant for the overall percentage of correct responses, *t* = −2.284, df = 18, p<.05, and for expressive melodies, *t* = −2.538, df = 18, p<.05, but not for deadpan melodies, *t* = −1.827, df = 18, p = .08.

## Discussion

### Passive Phrase Boundary Processing

#### P2

As Figure 2 shows, automatic stimulus identification was characterized by a large fronto-central P2 component. The P2 is commonly associated with stimulus identification and evaluation and has been shown to be sensitive to stimulus parameters such as intensity, pitch, and IOI [4]. In addition, the P2 is considered an index of neural plasticity and learning [25],[26]. In our experiment, the P2 was elicited in all conditions during passive listening. Expressive timing did not significantly affect the P2 amplitude. The finding suggests that the P2 reflects sensory encoding of stimulus features during passive exposure rather than higher-level stimulus classification. Although previous studies [13],[15] suggested that the P2 amplitude to phrase boundaries in music may be modulated by top-down processes, it is likely that top-down mechanisms require conscious processing to act upon stimulus identification.

#### P3a-Like Response

When the phrase boundary was marked by both a pitch leap and preceding timing information, a P3a-like response occurred. This deflection was fully absent when the melodies did contain timing variations but no additional pitch information at the phrase boundary. It is possible that this effect was simply due to the sensory processing of the pitch leap. However, if this was the case, we should have found a similar effect for the deadpan melodies. In the deadpan condition, the ERPs to phrase boundaries containing a pitch leap did not differ from those without a pitch leap. Thus, we suspect that the positive response in the ERP for expressive melodies containing a pitch leap at the boundary reflects some higher-order mechanism that could be related to expectation and attention allocation mechanisms.

There is ample evidence that the extraction of auditory temporal information creates expectations about the occurrence of sound objects [27],[28] (for a review of predictive processes in music cognition, see [29]). The violation of temporal expectations in music has been shown to be reflected in the ERP (e.g., [30],[31]). Assuming that expressive timing prepares the listener for an upcoming important event and therefore generates expectations, it is possible that the positive response in the ERP found in our study reflects the confirmation of the expectation generated by the preceding timing context.

Alternatively, our effect may be due to the allocation of attention towards the beginning of the new phrase. This idea is supported by the P3a-like frontally maximal distribution of the response. The P3a has been repeatedly linked to automatic attention allocation towards novel events (for a review, see [32]). In line, the beginning of the second phrase might have been automatically classified as such a novel event. It is remarkable that this effect occurred when the participants did not attend to the musical material. As has been shown previously, the P3a can be elicited during passive stimulus processing [12]. In such cases, the amplitude is smaller compared to active tasks. This finding not only suggests that the extraction of subtle timing variations happens implicitly. Importantly, it also shows that the extraction of timing information triggers attention mechanisms that facilitate the recognition and processing of relevant auditory objects. Further support for this assumption comes from the fact that the ERPs did not differ between deadpan melodies with pitch leap and deadpan melodies without pitch leap. It seems that, because of the lack of timing information signaling a possible occurrence of a new phrase, no expectations were generated and consequently no attention resources were allocated to the phrase boundary.

It is less likely that the effect was due to phrase closure processes as reflected by the music CPS. The music CPS shows a centro-parietal distribution and has so far only been reported during active music listening.

### Active Phrase Boundary Processing

#### P2

When participants attended to the melodies and performed a pitch-leap detection task, a clear P2 was elicited to non-target stimuli, hence, to the onset of the second phrase that was not marked by a pitch leap. The amplitude of the P2 was larger when the phrase boundary was preceded by expressive timing cues. In previous studies [13],[15] a significantly reduced P2 component for unphrased melodies compared to phrase boundaries that were preceded by a pause was found. Here, it is possible that the effect partially also reflected the recovery of neural populations during the pause. In contrast, in our study, this effect cannot be attributed to recovery of neural populations because the IOI was identical for both the deadpan and the expressive condition. It was also shown that the P2 amplitude was influenced by harmonic closure, preceding pause length and musical expertise [15] supporting the notion that the P2 is generally sensitive to higher order cognitive processes. In line, our findings support the assumption that the P2 is sensitive to top-down processes during attentive phrase boundary processing. Specifically, the extraction of global timing information seems to act upon stimulus identification.

#### N2b/p3a

During the active task, a large N2b component was elicited in response to phrase boundaries marked by the pitch leap. Because the pitch leap served as a target stimulus, the elicitation of the N2b was expected. The amplitude of the N2b was reduced in the expressive compared to deadpan condition.

Previously it was found that the N2b amplitude elicited by deviant pitch targets was larger when the degree of deviance increased [11]. Here, the N2b seemed to reflect the ease with which targets could be discriminated from the sound stream. Alternatively, there is also support for the assumption that the negative responses in the ERP around 200 ms are related to degree of expectedness [33],[34]. In line, it was shown that timbre changes serving as targets embedded in melodies elicited a clear N2b that was sensitive to the position of the timbre change within the melody [10]. For the earliest occurrence of the timbre change, the N2b amplitude was largest compared to later occurrences. That is, the N2b was more pronounced when the change was less expected. We assume that the N2b amplitude found in our study in response to target phrase boundaries was similarly affected by the degree of expectedness. The pitch leap within deadpan melodies was less expected than the pitch leap within expressive melodies, because timing already indicated its possible occurrence.

The N2b was followed by a P3a with fronto-central maximum. The P3a to target stimuli is believed to reflect activity within the frontal lobe that is affected by attention and task demands [12]. More attention resources are needed to perform difficult tasks. This allocation of resources is reflected in the P3a amplitude which is smaller when the task is demanding compared to less demanding tasks. The P3a amplitude was smaller for deadpan compared to expressive melodies in response to the target phrase boundary. This finding implies that the recognition of the phrase boundary was more difficult within the deadpan melodies compared to when the beginning of the new phrase could already be predicted on grounds of the preceding temporal information.

Together, the N2b-P3a complex seems to reflect the time course and strength of those processes underlying expectancy violation/confirmation and the resulting allocation of attention towards the target stimulus. The interaction between expectation and attention seem to provide the basis for conscious recognition of phrase boundaries in music.

#### Music Cps

The ERPs recorded to the processing of phrase boundaries in our study did not show any evidence for the previously found music CPS [13],[14],[15]. This equivocal finding might be due to the different cues used to mark the upcoming phrase boundary. Pause length and length of the final note of the first phrase were found to be the most important indices for the elicitation of the CPS [15]. These cues appear only shortly before the onset of the second phrase. Contrary, the expressive timing patterns as used in our experiment provided more global information about the phrase structure because the tempo was progressively diminished starting from the middle of the first phrase to its end. It could be speculated that closure processes as reflected by the CPS are based on the extraction of cues immediately preceding a phrase boundary without incorporating a broader auditory context. Such a local restriction, however, seems not to be supported by findings indicating that temporal processing in music relies on global patterns processing rather than on the identification of individual musical events [36],[37].

Alternatively, expressive timing information alone might not have been sufficient to build up a strong impression of phrase closure that would exceed the neural threshold for the elicitation of the CPS. The processes underlying the generation of the music CPS seem to be modulated by a variety of acoustic cues (e.g., harmonic function, temporal cues) [15]. Moreover, in contrast to previous studies [13],[14],[15], the melodies in our study did not contain any tonal information regarding the continuation or closure of a phrase. Instead, they consisted of equal interval steps that followed an ascending or descending order. Because musical phrase boundaries are often marked by a pitch fall [38], it is likely that this specific structure led to an expectation of continuation in the respective direction that was stronger than the expectation of the closure of a phrase as indicated by the temporal information. We therefore go further to suggest that for the CPS to occur, different acoustic cues need to be integrated to form a strong impression of closure. We assume that the combination of tonal information and expressive timing cues together indicating the closure of a phrase would be reflected in the CPS. This assumption, however, needs to be tested in future experiments.

The CPS seems to be dependent on musical expertise [15]. Contrasting brain responses of musicians and laymen, the authors found a clear CPS reflecting phrase closure processes only in music experts. Recent evidence suggests that the music CPS is also elicited in non-musicians [35]. In our study, the lack of the CPS, however, cannot be explained by the musical background of our participants, because neither musicians nor laymen showed this specific response.

### Differences Between Musicians And Laymen

During the passive session, the brain responses to the phrase boundary did not differ between musicians and laymen. It is possible that neither group treated the melodies as having phrase constituents during the passive listening condition and that we therefore did not find a difference between musicians and laymen. In the active condition, there was a clear N2b and P3a response to the pitch leap inserted in the melodies in both groups. That is, musicians and laymen showed similar response patterns in the ERP when they were forced to actively process the phrase boundary. When participants attended to the melodies, however, the N2b occurred earlier in musicians compared to laymen indicating that the detection of the phrase boundary was faster in the music expert group. Musicians also showed a larger P3a to the phrase boundary compared to laymen for the deadpan melodies. The finding suggests that the pitch leap detection task in our experiment was more demanding for laymen than for musicians when timing cues signaling the phrase boundary were lacking. Interestingly, there was no difference in P3a amplitude between laymen and musicians for expressive melodies. This finding suggests that the extraction of expressive timing cues facilitates the processing of subsequent auditory events through a process of increased attention allocation towards the anticipated sound object, especially when pitch discrimination abilities are not as precise as in trained musicians.

The detection accuracy for the pitch leap detection task was high in both musicians and laymen. Because of this ceiling effect, it was not possible to test for correlations between behavioral and ERP responses. Nevertheless, it would be very interesting to investigate the relationship between detection accuracy and neural responses in more detail. This could be addressed in future studies by manipulating the pitch leap so that the difficulty level spans an even greater range of intervals than used in the present paradigm.

### Conclusion

The present study extends current knowledge about the neural mechanism underlying the sequential organization of speech and music by demonstrating that expressive timing facilitates the recognition of phrase boundaries in music. We show that temporal information is extracted on a global level to generate expectations and to activate the allocation of attention towards a specific musical event.

It might be argued that our experimental stimuli were less ecological valid than the music excerpts used previously. This might also explain why we did not find the proposed music CPS component. Notwithstanding, the present melodies allowed us to strictly control for possible effects of harmonic context, inter-onset interval, tempo, and melodic progression. Thus, we are able to solely attribute the results to the extraction of temporal variations as induced by expressive timing.

It might be questioned whether phrasing patterns as used in Western tonal music may be similarly applicable to atonal melodies because phrasing has been shown to be determined by the harmonic structure of music [39]. Beyond harmony, however, rhythmic grouping [40] and metrical structure [38] serve the organization of music into phrases. More fundamentally, general principles of perception as formulated already by Gestalt theory in the beginning of the 20^th^ century [20] may account for the grouping of auditory information such as music. For instance, the principle of proximity describes how tones that are close in pitch, timbre or dynamics are grouped together [1],[38]. Consequently, the occurrence of a pitch leap is likely to be processed as the beginning of a new auditory group. The expressive timing patterns used in our paradigm are in line with the grouping structure of auditory information as predicted by Gestalt-principles. The application of phrasing patterns as used in Western tonal music therefore seems justified.

In the present study, we carefully controlled for possible effects of tonal and metrical expectations by using atonal melodies and by inserting the pitch leap at different positions within the melodies respectively. We also controlled for effects of tempo by using three different tempi equally distributed over the melodies. Importantly, we also assured that the processing of the pitch leap was not affected by the duration of the event directly preceding the pitch leap. That is, the difference in behavioral and brain responses found between deadpan and expressive melodies cannot be explained by the lengthening of a single event preceding the phrase boundary. Taken together, this careful design allows us to attribute the difference between the two conditions to the experimental manipulation of the timing patterns applied to the first phrases of the melodies. That is, expressive timing and, hence, the phrase structure of the preceding context seems to explain the difference in the neural processing of the pitch leap.

Timing is only one aspect that is prone to subtle variations during music performance. Loudness, pitch, timbre and other acoustic cues also contribute to a performances’ micro-structure. It would therefore be important to study the effects of individual parameters and their combinations on the neural processing of phrase boundaries to identify the relative contribution of each acoustic cue. Another important aspect that should be addressed in future studies is the role of the individual performer. Similar to individual speaker variations, music performers do have their own strategy to interpret the musical structure. Some might be more successful in conveying the structure in such a manner that it matches the listeners’ sensory and cognitive strategies than others. We suspect that compatibility between performers’ expression and listeners’ perception could account for preferences for one performer to the other.

Our results revealed that expressive timing modulates the processing of phrase boundaries even when the music is ignored. This is an interesting finding that emphasizes the automaticity of auditory sound organization. It provides further evidence for the notion that auditory information is implicitly but constantly being evaluated in terms of its significance. From a biological perspective, the ability to passively and actively extract important auditory events from sound streams such as music or speech is crucial for an organism to act in and to react to its environment in an appropriate manner.
